# Characterization and Valorization of Maize Landraces from Aosta Valley

**DOI:** 10.3390/plants12142674

**Published:** 2023-07-17

**Authors:** Alessandra Lezzi, Lorenzo Stagnati, Francesca Madormo, Denise Chabloz, Alessandra Lanubile, Marilisa Letey, Adriano Marocco, Mauro Bassignana, Matteo Busconi

**Affiliations:** 1 Dipartimento di Scienze delle Produzioni Vegetali Sostenibili, Università Cattolica del Sacro Cuore, Via Emilia Parmense 84, 29122 Piacenza, Italy; 2 Centro di Ricerca BioDNA, Università Cattolica del Sacro Cuore, Via Emilia Parmense 84, 29122 Piacenza, Italy; 3 Institut Agricole Régional, Reg. La Rochère 1/A, 11100 Aosta, Italy

**Keywords:** maize landraces, agrobiodiversity, Aosta valley, genetic characterization, genetic resources

## Abstract

While there is a rich collection of maize germplasm from Italy, it lacks genetic resources from the Aosta Valley, an isolated mountain region where landraces have been preserved in the absence of modern germplasm introductions. These local materials, which are still cultivated mainly at household level, can have high importance from a genetic and historical point of view. In the present study, five landraces named, after the collecting sites, Arnad, Arnad-Crest, Châtillon, Entrebin and Perloz, were sampled in Aosta Valley and subjected to historic, morphologic and genetic characterization. This study provided evidence for the landraces’ long presence in Aosta Valley, a significant genetic variability and differentiation among the investigated landraces. Globally, 67 different alleles were detected ranging from 4 for markers *phi127* and *p-bnlg176* to 10 for *phi031*, with a mean of 6.7 alleles per locus. Observed heterozygosity levels were comprised from 0.16 to 0.51 and are generalkly lower than expected heterozigosity supporting fixation at some loci. STRUCTURE analysis revealed clear separation between accessions revealing the presence of four ancestral populations. This may be explained by the long reproductive isolation experienced by these materials. Finally, morphological observations confirm the high diversity between landraces revealing that they generally have flint kernels, variable color from yellow to dark red (Châtillon) while Perloz showed kernels with an apical beak. The present work confirms the importance of mountain areas in conserving biodiversity and increases the rich Italian maize germplasm with materials well adapted to marginal areas. Such new genetic variability may be used to breed new materials for more resilient agriculture.

## 1. Introduction

Maize, *Zea mays* L., represents the leading cereal crop in terms of production and yield per hectare in Italy [1].

Looking back in time, the introduction of maize in Italy dates back to 1493 when Christopher Columbus brought this cereal back from his travels [2]. From that moment, its cultivation began to grow more and more leading in the centuries to the selection of local varieties (landraces) perfectly adapted to the different microclimatic areas of the Italian territory [2,3]. Landraces are defined as dynamic populations of genetically diverse individuals well adapted to local conditions, associated with traditional agricultural practices and local history; moreover, they can be considered the ultimate expression of crop domestication [4].

In Italy, maize landraces have been extensively grown until the mid of the twentieth century, when the cultivation of hybrid took place due to their significant agronomic performances. During the years 1949–1950 a formal investigation started to characterize maize cultivation and in 1954 a project for the sampling of Italian maize landraces was activated resulting in the preliminary collection of 562 different accessions. In subsequent years other materials have been collected, increasing the number of landraces currently available in ex situ collections [2,5,6].

Interestingly, of the 562 landraces sampled in 1954 or later, none was collected in Aosta Valley being the only Italian region which did not contribute to the maize sampling [2]. While there is no explanation for the absence of any germplasm coming from this region, a map of maize cultivation in the first half of the twentieth century shows that in Aosta Valley maize was, more or less, widely cultivated [7].

Presumably, maize was introduced in Aosta Valley in the 18th century [8]. Although there is no certain date and place attesting to the introduction of the crop in Aosta Valley, its food use was known since the beginning of the century [9] and its cultivation is documented in 1779 in Donnas [10] and 1785 in Montjovet [11]. Then, the cultivation spread along the central valley, in particular near Aosta [11]. For about two centuries maize became the main source of food for many families in the region [12].

In many cases, suggestions to improve Italian agriculture in the first half of twentieth century, were often addressed to reduce maize cultivation in all the areas where maize could find unfavorable conditions as in the hills or mountain valleys [13]. In this frame, Aosta Valley was not an exception because suggestion on this direction were given by several authors even before those of Zapparoli [13].

The *Comice Agricole de l’Arrondissement d’Aoste*, an association committed to the promotion of agricultural production, advised against the cultivation of maize in the Middle and Upper Valley as it was not profitable [14] and Argentier [8], emphasizing the high manpower required for the crop, recommended to replace it with more productive ones. Following the inauguration of the Chivasso-Aosta railway in 1886, Vescoz [11] underlined that the cultivation of maize had progressively decreased in Aosta Valley due to the competition from maize coming from Piedmont.

Historical records outline maize cultivation on 12–13 thousand hectares in the period 1939–1944, data are missing in 1945 and 1946 while from 1947 to 1956 maize cultivation decreased progressively from 740 to 696 hectares.

Even after the introduction of modern varieties, intensive maize cultivation in Aosta Valley never started, not even in the most favorable areas: since regional agriculture is mainly devoted to the production of hay, maize and other relevant cereal species are rarely present. In 1957, maize hybrids were introduced starting on a surface of 5 hectares while traditional landraces, recorded as “Nostrano”, were cultivated in 685 hectares. Hybrids reached a peak of 152 ha in 1970 followed by a drop to 46 ha in 1971. From that time hybrid cultivation decreased to the actual 6 ha. Official records have the distinction between hybrids and landraces from 1957 to 1974 but from 1973 landrace cultivation seem to be absent on relevant surface remaining at home level for family purposes. The absence of an intensive agricultural system and the peculiar morphology of the region allowed the prosecution of a more traditional farming system, mostly at home level which is still very practiced. These factors allowed the conservation of several landraces, genetic resources that are a lifeline against the erosion of biodiversity [15].

Therefore, this region can represent an interesting basin in which to investigate the presence of relict landraces and local populations of agricultural crops which are still in situ/*on farm* or to characterize what is preserved ex situ in collection as the one held at the Institut Agricole Régional (IAR) in Aosta (Italy). In the framework of biodiversity conservation, the importance of mountain areas has been addressed by several authors [16,17]. Mountains are essential areas to counteract the general loss of agrobiodiversity, which in turn represents a valuable opportunity to create small agri-food chains thus enhancing the resilience and economy of marginal areas [4]. In the case of maize landraces, interesting discoveries of several unique landraces have been completed in mountain areas even in the recent past [3,5,18,19].

In the context of a climate-changing agricultural scenario, there is an urgent need to discover new sources of adaptation to biotic and environmental stresses that may contribute to crop breeding aimed at maintaining productivity and reducing inputs. To address this objective, landraces can be valuable resources since they are characterized by high genetic variability and they can present favorable alleles for increasing the adaptation to harsh conditions and challenging environments that may have been selected through natural and human pressure [15,20].

Germplasm resources can be characterized at different levels on the base of the objective of biodiversity preservation. For scientific purposes, genetic and morphological characterizations are the most important, with genetic studies being essential to understand the conservation status, risk of genetic erosion and the importance to preserve that genetic material for present or possible future uses.

Genetic characterization of crop landraces, particularly maize, is performed by means of molecular markers; among the many that are currently available, the two most used are Single Nucleotide Polymorphisms (SNPs) and Simple Sequence Repeats or Microsatellites (SSRs). In maize, SSRs have been used to investigate the genetic biodiversity and structure of many germplasm collections constituted from few to hundreds of accessions [3,21,22,23,24,25,26].

Trying to fill the gap of knowledge on traditional maize germplasm, the present research was aimed at the genetic and morphological characterization of maize landraces from Aosta Valley preserved ex-situ at the Institut Agricole Régional. In the study, five local varieties—named after the sampling location as it follows: Arnad, Arnad-Crest, Châtillon, Entrebin and Perloz—have been subjected to historical, morphological and genetic characterization.

## 2. Results and Discussion

### 2.1. Historical and Phenotypic Characterization of Maize Landraces

**Figure 1:**
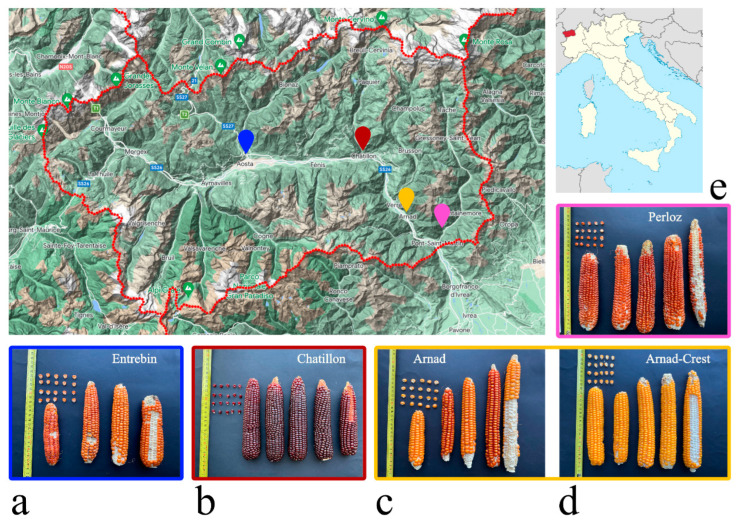
Sampling location ad ear morphology of the five maize landraces. (**a**) Entrebin; (**b**) Châtillon; (**c**) Arnad; (**d**) Arnad-Crest and (**e**) Perloz. On the map, the location of Entrebin is reported in blue, Châtillon is reported in red, Arnad and Arnad-Crest in yellow, finally Perloz is reported in pink.

Landrace Arnad is characterized by tall plants, on average from 275 to 300 cm tall, presenting as mean value of the population a small ear/plant insertion ratio. Leaves characters are generally variable; the majority of plants have a semi-erect or horizontal leaf attitude while the insertion angle between leaf and stalk is between 25°–50°. Anthesis and silking occurred at 72 and 78 DAS, respectively. Tassels are characterized by a very high number of medium/long primary branches, i.e., 18; the angle of insertion between the main axis and primary branches is variable, mainly between 50° and 75°, with horizontal and drooping attitude finally; tassels’ length varies from 31 to over 50 cm. Concerning the tassel component, the glume ring is uniform and of absent-weak pigmentation, glumes are variable but mainly of absent or weak pigmentation while anthers pigmentation may vary from absent-very weak to strong, while silks range from absent-very weak to medium pigmentation. Ears are of slightly conical shape of medium-long length (19–24 cm) presenting from 8 to 10 rows of flint or flint-like kernels which vary from yellow-orange to orange color and are inserted on a white cob (Figure 1). Ear and kernel morphology of Arnad resembles that of landraces classified in the Ottofile Ratial Complex as described by Brandolini and Brandolini [27].

Landrace Arnad–Crest is characterized by very tall plants that are able to reach and pass 300–320 with a medium (46–50%) ear/plant insertion ratio as population average. Anthesis and silking occurred at 80 and 86 DAS, respectively. Leaves are generally of horizontal-dropping attitude while the insertion angle between leaf and stalk is highly variable and comprised between 25°–75°. Tassels are characterized by a very high number of long/very long primary branches of erect semi-erect attitude and medium-high insertion angle. Focusing on Tassel’s traits, high variability was observed. The glume ring can be either of absent pigmentation as well as variably pigmented, glumes have absent of weak pigmentation while anthers have absent, weak or medium pigmentation. Silks are, mainly, of absent-very weak pigmentation.

Ears are of slightly conical shape of medium-long length (19–24 cm) presenting from 10 to 16 rows of flint-like kernels which vary from yellow to yellow-orange color and are inserted on a white cob (Figure 1). Ear and kernel morphology of Arnad-Crest resembles that of landraces classified in the Ottofile Derivati Ratial Complex as described by Brandolini and Brandolini [27].

For Arnad and Arnad-Crest, the historical study was not carried out due to a lack of information.

Landrace Châtillon is characterized by a medium height of 265 cm and a short ear/plant insertion ratio. Anthesis and silking occurred at 70 and 75 DAS, respectively. Leaf attitude is semi-erect/horizontal while the angle of insertion between leaf and stalk is mainly between 50°–75°. Tassels are characterized by a very high number (24) of primary branches of variable attitude from semi-erect to dropping. The ring at the base of the tassel’s glumes is generally uniform of absent-very weak pigmentation, glumes and anthers are variable from absent-very weak to medium pigmentation. Silk pigmentation is absent-very weak. Ears are of slightly-conical shape bearing from 12 to 16, sometimes 18 rows of flint and flint-like kernels with a characteristic dark red color; also the cob is pigmented (faint-strong pigmentation, Figure 1).

Châtillon was given to the current custodian farmer fifteen years ago by an acquaintance who cultivated maize to produce polenta in Châtillon. It was not possible to go further back in time with information. The custodian farmer has been cultivating Châtillon according to the usual maize cultivation techniques, placing the crop in the rotation. The seeds are self-produced year after year. For this purpose, the earliest ears are chosen, which have a larger size and a uniform color. This practice has made it possible to improve the crop over time. Once the ears have been harvested, they are left to dry, tied and hung in an airy place, then shelled once dried. As per tradition, the current custodian farmer, in addition to using maize flour for polenta, uses it to produce “*lasole*”, a sort of semolina obtained with Châtillon flour and milk.

Landrace Entrebin is characterized by plants of 250 cm as medium height and with a small ear/plant insertion ratio. Anthesis and silking occurred at 66 and 70 DAS, respectively. Leaves are both of horizontal and dropping attitude while the insertion angle between leaf and stalk is mainly between 25°–50°. Tassels are characterized by a very high number of primary branches with an erect to horizontal, rarely dropping, attitude. Tassels’ length ranges from 31 to 50 cm. The glume ring is uniform of absent-very weak pigmentation, glumes are variably pigmented (from absent to medium) while anthers are of absent-very weak or weak pigmentation. Silk pigmentation is variable with the majority of plants having weak or medium pigmentation (pink and red) but green silks are present as well. The ears’ shape is slightly conical, between 10–16 rows of orange or red-orange flint kernels inserted on a, generally, white cob (Figure 1).

**Figure 2:**
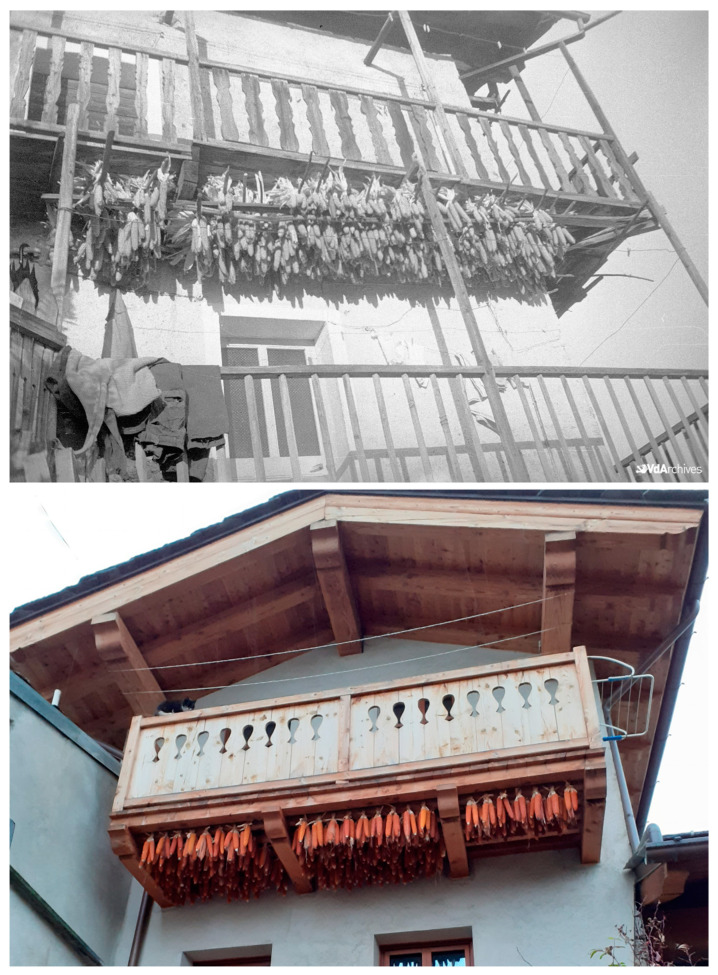
Historical (1952; Photographer Octave Bérard. Regione Autonoma Valle d’Aosta, BREL Archive—Collection Bérard) and present (2021; Archive IAR) traditional drying of ears at the family who preserved Entrebin maize landrace.

Entrebin cultivation dates back to the early 1900s, when the paternal grandmother of the current grower brought the seeds from Signayes (AO) as a dowry. Today, as in the past, the cultivation of maize by the custodian farmer’s family is mainly intended to produce flour and only partially reserved for animal feed. Between the 60s and 70s of the last century, the mill where most of the families in Aosta went for milling closed, and the Entrebin maize was exclusively used for cattle, until the purchase of a small family mill in the early 1980s. The custodian farmer cultivates it according to traditional maize cultivation techniques, placing the crop in rotation and self-producing the seeds. In the period preceding the harvest, the ears from which the seeds will be taken for the following year are chosen, selected from the healthiest and precocious plants. The seed ears are harvested a few days before the harvest time and are left to dry, as in the past, hanging from the balcony of the family house. At the beginning of spring, they are shelled by discarding the ends that have smaller seeds and keeping the central part. Even the remaining part of the harvest is left to dry hanging from the balcony of the house. As per tradition, the ears are tied together in threes, making a knot with the bracts (Figure 2).

Landrace Perloz is characterized by tall plants (280 cm as medium value) and a small ear/plant insertion ratio. Anthesis and silking occurred at 73 and 77 DAS, respectively. Leaf attitude is semi-erect/horizontal while the angle of insertion between leaf and stalk is mainly between 25°–50°. Tassels length is comprised between 31–50 cm classifying tassels as medium–long. The number of primary branches is high or very high and they are characterized by horizontal or dropping attitude.

The glume ring is generally uniform of absent-very weak pigmentation, both glumes and anthers are of absent-very weak or weak pigmentation sometimes medium while rarely strong. Silks are of variable pigmentation, from absent-very weak to medium.

Ears are of slightly conical shape and present between, mainly, 14 to 16 rows of flint yellow-orange orange-red kernels on a white cob (Figure 1). Moreover, kernels of this landrace show a characteristic beak on the top. For Perloz, the historical study was not carried out due to a lack of information.

The landrace collection here investigated is characterized by accessions of flint/flint-like kernels, whose color varies from yellow to red with the exception of Châtillon, which is the only accession with dark-red ears, a character not common in landraces sampled in 1954 [27] but very often present in landraces of recent collection [3,18,19,25,26]. Vegetative cycles are variable, the earlier landrace is Entrebin, which is the one maintained at the highest altitude, while the others are grown on the valley floor where temperatures are higher allowing longer cycles; probably the windy climate of Aosta Valley helps the good drying even of later landraces, such as Arnad-Crest. Morphological traits such as leaf insertion angle and attitude, as well as the tassel type, strongly support the nature of the old landrace of these materials. Indeed, tassel size has dramatically reduced from open pollinated varieties to modern maize hybrids in the last decades. Big tassels are interpreted as a potentially valuable trait in populations with open pollination, heterozygosity, and attendant variation in silking dates [28]. Concerning leaf attitude and angle, upright leaves have been correlated to high yield because of tolerance to higher plant densities. Modern maize selection strongly prefers materials with upright leaves [29,30,31]. Interestingly, only the landrace Perloz can be classified in the group of “Rostrata” having pointed-beaked kernels. This particular trait is very common in mountain maize landraces since it is associated to better drying and storage ability of the kernels [4]. Intra-population variability was noticed for characters that are not considered by farmers for selection, i.e., tassel traits, while farmers’ relevant traits as ear shape and color appear to be uniform or fixed. It is reported that farmers apply strong morphological selection of few traits and, even in case of strong genetic flow between materials, they are able to maintain phenotypic distinction of landraces [32].

### 2.2. Genetic Characterization of the Accessions

Markers data for all 92 samples were collected and analyzed to investigate main population parameters. Globally, 67 different alleles were detected ranging from 4 for markers *phi127* and *p-bnlg176* to 10 for *phi031*, with a mean of 6.7 alleles per locus (Table 1).

The high number of detected alleles may derive from the reproductive isolation that the landraces under study have experienced in the last decades in the context of a region characterized by high mountains, and very limited maize cultivation in small and highly isolated fields.

All loci were polymorphic in the populations with the exception of *phi076* in Arnad, *umc1786* and *umc1914* in Châtillon. Moreover, in some cases, the presence of only two alleles or a prevalent allele has been noticed (i.e., *phi031*, *umc1401* in Entrebin, *phi076* in Perloz, *umc1327* in Entrebin and Perloz, *phi084* in Entrebin and Châtillon and *umc1786*, *p-bnlg176* in Arnad-Crest and *phi127* in all populations).

This may be a consequence of the isolation experienced by landraces and/or of a moment of reduced cultivation that occurred at any time in the last decades. At a household scale of cultivation, it is common to grow few plants and save seeds from a very few number of ears responding to a particular ideotype. It cannot be excluded that some alleles went lost during that time. Similar studies on maize landraces report both high levels of polymorphism at collection/locus as well as the presence of monomorphic loci in some landraces [3,21,22,24,25,26] as a possible consequence of selection for some interesting character particularly appreciated by local growers.

This collection seems to be particularly rich in private alleles as reported in Table 2: 4 private alleles have been detected in Entrebin (*phi031*, *umc1786*, *umc1941*), Châtillon (*phi076*, *umc1401*, *phi031*, *umc1075*), Arnad (*umc1401*, *phi031*); 6 private alleles in Perloz (*phi127*, *phi031*, *phi084*, *umc1786*, *umc1941*); 8 private alleles in Arnad-Crest (*phi076*, *umc1401*, *phi127*, *phi031*).

The high percentage of private alleles over total alleles (39%) may reflect the reproductive isolation of these landraces.

The number of observed alleles (Na) ranged from a minimum of 2.20 of *phi127* to a maximum of 3.40 of *phi076*, *umc1401*, *phi031*, *umc1075*, *phi084*, and *umc1941* at locus level; while, at the population level, it ranged from 2.40 for Châtillon to 3.80 for Arnad-Crest. The number of expected alleles (Ne) was lower without any exception than Na spanning: at locus level from 1.39 for *phi127* to 2.49 for *umc1075*; at landrace level from 1.51 to 2.47 in Châtillon and Arnad-Crest, respectively (Table 1). To better characterize landraces diversity Shannon’s index (I) was used, which was found to be, on average, equal to 0.78 ± 0.16 at locus level, and 0.78 ± 0.21 at landrace level (Table 1). Mean values of the observed heterozygosity (Ho) were equal to 0.40 ± 0.15 at the locus level and 0.40 ± 0.14 at the landrace level; whereas the unbiased expected heterozygosity (uHe) mean values were equal to 0.46 ± 0.09 at locus level and 0.46 ± 0.12 at landrace level. These latter findings suggest fixation at the following loci: *phi076*, *umc1075*, *umc1327* and *p-bnlg176*. Instead, at the landrace level, the differences in heterozygosity are more limited. Moreover, the inbreeding coefficient F_IS_ had average values of 0.12 ± 0.33 and 0.16 ± 0.13; this and the lack of heterozygosity confirm the presence of fixed or selected loci as previously seen. F_IS_ gives an indication of the reproductive history of the population, with values close to 0 indicating a random mating system that is close to the Hardy–Weinberg equilibrium, as observed for Perloz which is slightly outbred. Positive F_IS_ values mean a certain level of inbreeding up to 1 for completely inbred genotypes. Some level of inbreeding is present in all landraces, except Perloz, though it is more evident for some loci (*phi076*, *umc1075*, *umc1327*, *p-bnlg176* and *umc1941*) while the other loci are closed to Hardy-Weinberg equilibrium or outbred (Table 1). F_ST_ is a measure of differentiation and it was equal to 0.32, suggesting that the landraces under study have a very high of differentiation, of which 32% is found to be between varieties [33]. Average PIC value was 0.63 ranging from 0.48 of *phi127* and *p-bnlg176* to 0.74 of *phi031* and *umc1786*. PIC is one of the indicators of marker quality in genetic studies and corresponds to its ability to detect the polymorphism among individuals of a population, and the higher that capacity, the greater its value. For co-dominant markers, PIC value can range from 0 (monomorphic) to 1 (several alleles of equal frequency) and markers whose PIC value is greater than 0.5 are considered to be very informative [34]. The markers used to characterize the present landrace collection are adequate and can be considered as highly informative.

### 2.3. Cluster Analysis and Phylogenetic Tree

**Figure 3:**
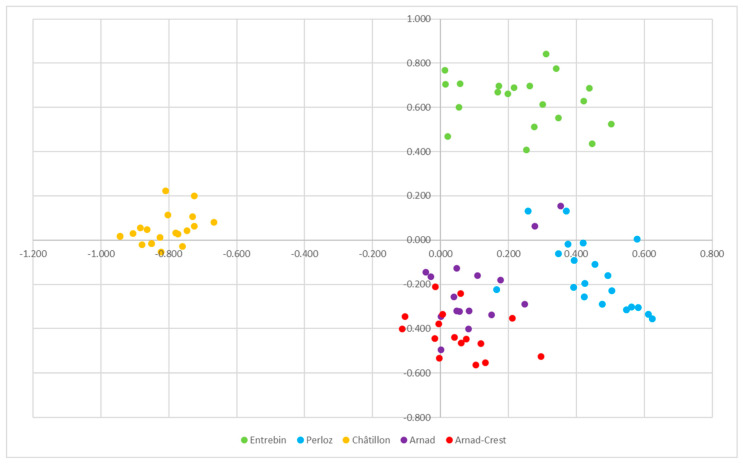
Principal Coordinates Analysis (PCoA): coordinate 1 vs. coordinate 2 of the 92 samples characterized by the 10 SSR set.

Principal Coordinate Analysis (PCoA) provided a good separation of the landraces since the two first principal components account for 21.53% and 14.68% of genotypic variability. The clear separation resulted for landrace Châtillon, Entrebin and Perloz while Arnad and Arnad-Crest, even if easy to identify, were generally overlapped as reported in Figure 3 and Supplementary Material S1. Such good separation, as previously mentioned, may derive from the long reproductive isolation of the landraces as supported also by the F_ST_ and an interesting number of private alleles. Good levels of differentiation have been reported for landraces maintained for decades in situ under reproductive isolation in Emilia Romagna, while traditional germplasm sampled in the ‘50s did not show clear differentiation [3,26]. If the PCoA is computed according to landrace names the variation explained by the two first principal components accounts for 40.78% and 27.81%, the most relevant finding is a more evident separation among Arnad and Arnad-Crest which remain very closed (Supplementary Figure S1).

**Figure 4:**
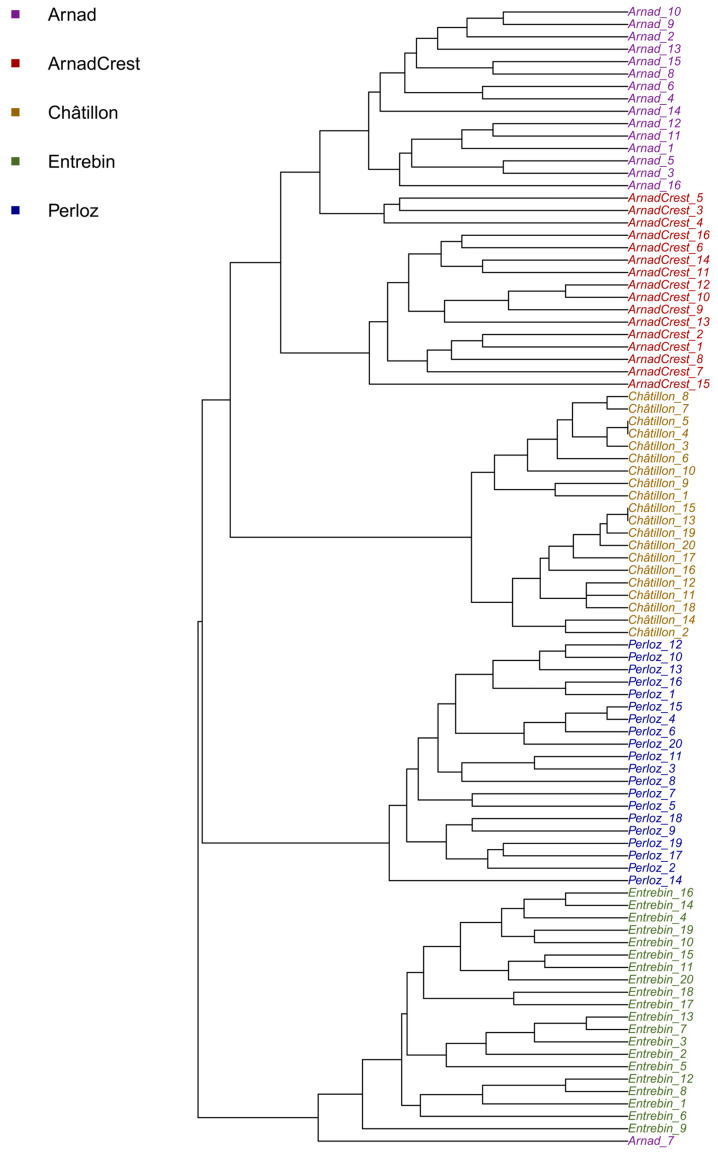
Phylogenetic tree of the 92 individuals of the five maize landraces from Aosta Valley.

The UPGMA phylogenetic tree of individuals shown in Figure 4 supports the PCoA results previously shown: indeed, it revealed a clear clusterization of samples.

From the tree, it can be seen the evident differentiation of all the five landraces with Arnad and Arnad-Crest is more closely related since they depend on the same tree ramification, but still differentiated at the population level. These observations are supported also by the computation of Nei’s genetic distance (Table 3) and from pairwise F_ST_ (Table 4) which supported a high degree of differentiation between all landraces with the most closely related being Arnad and Arnad-Crest.

Similar observation and strong differentiation between landraces have been found by Stagnati et al. [3] when analyzing landraces conserved in situ under strong reproductive isolation for many decades, while, if landraces have been sampled in a context of strong landrace cultivation, the differentiation is not so clear [26].

### 2.4. Population Structure

**Figure 5:**
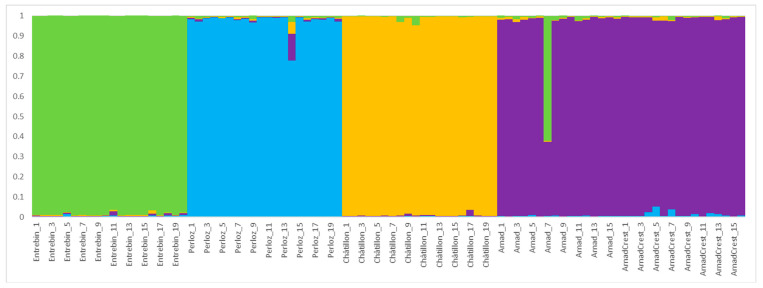
Population genetic structure at K = 4 of the 92 individuals of 5 maize accessions evaluated in the present study. Different colors correspond to different ancestral populations.

**Figure 6:**
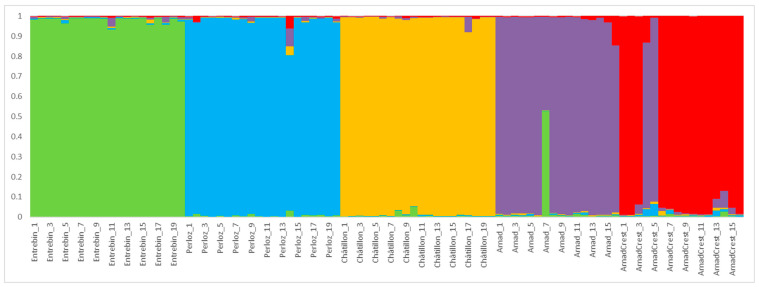
Population genetic structure at K = 5 of the 92 individuals of 5 maize accessions evaluated in the present study. Different colors correspond to different ancestral populations.

The analysis of population structure revealed two clear levels of differentiation. The first level of differentiation was found for K = 4 (ΔK = 1367.95) while the second at K = 5 (ΔK = 578.82). At K = 4, as shown in Figure 5, landraces as Entrebin, Perloz and Châtillon were clearly separated supporting an independent origin of these materials, almost all individuals (100% for Entrebin and Châtillon, 95% for Perloz) showed strong ancestry association (>90% of membership) to their cluster. Landraces Arnad and Arnad-Crest were highly associated with the same cluster with, respectively, 93% and 100% of individuals in the cluster. The main finding at K = 5, as reported in Figure 6, is the separation of Arnad and Arnad-Crest. Because of the high level of membership at K = 4, cluster association at K = 5 was evaluated at a stringent threshold (0.8) instead of 0.5 as reported by other researchers [24,26]. At K = 5 high levels of association were detected for Entrebin and Châtillon (100%) followed by 95%, 93% and 87% of individuals of Perloz, Arnad and Arnad-Crest, respectively. The only individual who can be considered as admixed, with a membership <50% to the landrace cluster is individual 7 of Arnad (Figure 6).

The results of structure analysis are consistent with the PCoA and phylogenetic tree (Figure 3 and Figure 4) delineating a situation of independent origin of the landraces which are clearly separated and share limited genetic composition. The partial relatedness of Arnad and Arnad-Crest, already hypothesized by PCoA, phylogenesis and Nei’s distance, is supported also by structure analysis. Moreover, these landraces have been sampled in the same municipality (Arnad). Thus, it is not possible to exclude, even if limited, the exchange of pollen. Considering morphology, Arnad landrace is a typical “Ottofile” while Arnad-Crest is more difficult to classify in morphological schemes. If these materials are related, it is possible to suppose that an Arnad strain fertilized an ancestor of the actual Arnad-Crest, as suggested by the phylogenetic tree and population structure analysis. Moreover, in Donnas, a municipality nearby Arnad, a maize population of composite origin, deriving from a recent cross of local maize and Piedmont strains of the type “Pignoletto”, is cultivated [19].

This strong population-cluster association and differentiation are very interesting if compared to similar works where different levels of admixture have been detected [3,21,24,26]. For maize landraces, it is reported that farmers perform directional selection based on a limited number of traits, which often are kernel type, color or particular ear shape. This directional selection causes a clear separation of landraces at the morphological level, even if there is an extensive gene flow between landraces [3,25,26,32].

Probably, the strong reproductive isolation, the almost complete absence of relevant maize cultivation in the last 60 years, and no, or limited, introduction of improved varieties have helped the maintenance of population distinctiveness. The gene flow of these landraces is lower with respect to other local populations of northern Italy sampled where landraces were widely cultivated [26] and more similar to materials that experienced isolation [3,24,25].

The highest gene flow (Table 1) is for Arnad-Crest thus supporting again a more recent and putatively complex origin than other landraces.

Landrace distinctiveness measured for this first set of maize landraces from Aosta Valley encourages the hope to find new accessions, still unknown up to now because hidden in the mountains of this alpine region.

## 3. Materials and Methods

### 3.1. Germplasm Acquisition and Historical Characterization

Landraces’ seeds used in this work were maintained in the germplasm collection held at IAR, Institut Agricole Régional (La Rochère AO), and they were named after the sampling location as follows: “Arnad”, “Arnad-Crest”, “Châtillon”, “Entrebin” and “Perloz”; sampling location is reported in Figure 1.

For each landrace, when it was possible to find information, a historical study was carried out. The methodology adopted integrates different techniques: interviews with custodian farmers, historical-bibliographic, historical-photographic documentation and direct observation of the current agricultural practices, conservation and use of maize.

### 3.2. Experimental Fields and Phenotypic Characterization

Landraces were grown in an experimental field located in Saint-Marcel, Aosta, Italy (45.738647 N, 7.440272 E) at the Agricultural demonstration center of Regione Autonoma Aosta Valley, sown the 6 May 2022. For each landrace, a plot constituted of four adjacent rows of 5 m in length and spaced 0.8 m apart was sown. Each row accounted for 25 seeds, one every 20 cm. The experimental field was managed according to standard agronomic practices for maize nurseries.

Landraces were reproduced according to a random-intermating system in order to avoid selfing and fresh seeds were stored in the germplasm collection held at the IAR and at the Department of Sustainable Crop Production of Università Cattolica del Sacro Cuore (Piacenza, Italy).

Phenotypic characterization was performed on 40 plants for each variety, according to the UPOV TP/2/3 protocol (International Union for the Protection of New Plant Varieties) in Saint-Marcel (AO) during the growing season. Anthesis and silking were recorded as Days After Sowing (DAS).

### 3.3. DNA Extraction and PCR Amplification

Young leaf tissues were sampled from the field of Piacenza at the V5 stage (fifth leaf). DNA was extracted with GenElute™ Plant Genomic DNA Miniprep Kit (Merck Life Science s.r.l., Darmstadt, Germany) following manufacturer instructions. The extracted DNA was then visualized and quantified on 1.0% agarose gel electrophoresis stained with Eurosafe nucleic acid stain (EuroClone, Pero, Italy).

Ten SSR markers previously reported for the characterization of Italian maize landraces [3,25,26] were used for DNA analysis. Detailed information on primer pairs is reported by Stagnati et al. [25]. PCR reactions were carried out using the following mixture: 20 ng of genomic DNA, 1× Reaction Buffer (2.5 µL 10× Reaction Buffer), 12 pmol dNTPs (0.3 µL of 10 mM stock), 4 pmol primer forward and reverse (0.4 µL of 10 µM), 1 U *Taq* polymerase (0.2 µL of 5 U/µL stock), and H_2_O to final volume of 25 µL. The PCR cycles were performed as reported by Stagnati et al. [25].

Fragments of different fluorescence and size were multiplexed and separated using an ABI 3130xl Genetic Analyzer sequencer (Applied Biosystems, Waltham, MA, USA). PCR fragment visualization and sizing were performed using GeneMapper software version 4.0 (Applied Biosystems).

### 3.4. Statistical Analysis

Detected alleles were analyzed with the GenAlEx6 software [35] to compute population statistics, analysis of molecular variance (AMOVA) and Principal Coordinates Analysis (PCoA). The Polymorphic Information Content (PIC) was calculated with PowerMarker software, version 3.25 [36].

A phylogenetic tree was constructed using the Unweighted Pair Group Method with the Arithmetic mean method applying the *upgma* function of the Phangorn package [37] starting from a genetic distance matrix calculated by the *meandistance.matrix* available in the polysat [38] package of the R software version 4.2.2.

The population structure of the maize collection was examined using a Bayesian clustering algorithm implemented in STRUCTURE v.2.3.4 [39]. The “admixture model” and the “correlated allele frequency model” were selected as suggested [24,39]. Ten independent replications were run for each level of K ranging from 2 to 10 with a burn-in of 2 × 10^5^ and 10^6^ Markov Chain Monte Carlo replications. The best estimation of K was selected according to the method of Evanno [24,40] Assignations were allotted according to Palumbo et al. [24].

## 4. Conclusions

For the first time, five maize landraces from Aosta Valley have been sampled and characterized. Genetic analysis revealed that these landraces present intra-population variability while high levels of differentiation exist between populations. Ancestry analysis revealed that three of the sampled landraces have an independent origin, while the remaining two are more related. The high genetic differentiation finds correspondence at the phenotypic level. Generally, these landraces have flint kernels, a variable color from yellow to dark red. The landrace Arnad is probably a representative of the Eight-rowed Flint ratial complex while the other accessions are of undetermined type. Interestingly, Perloz showed kernels with an apical beak, a common feature of mountain maize landraces, which suggests that this landrace belongs to the “Rostrata” group.

Overall, the present work confirms the importance of mountain areas in conserving biodiversity and increases the rich Italian maize germplasm with materials well adapted to marginal areas. Such new genetic variability may be used to breed new materials for more resilient agriculture.

## Figures and Tables

**Table 1:** Genetic parameters calculated according to the ten SSR and five landraces object of the study. Average number of observed alleles (Na), effective number of alleles (Ne) per locus, Shannon index (I), observed (Ho) and unbiased expected (uHe) heterozygosity, polymorphism information content (PIC) and Wright’s inbreeding coefficient FIS, FIT, FST and gene flow (Nm) are reported.

	Alleles	Na	Ne	I	Ho	uHe	PIC	F	F_IS_	F_IT_	F_ST_	Nm
***phi076***	10	3.40	2.21	0.76	0.18	0.41	0.72	0.65	0.57	0.74	0.39	0.40
***umc1401***	7	3.40	1.97	0.84	0.55	0.50	0.67	−0.11	−0.09	0.19	0.26	0.72
***phi127***	4	2.20	1.39	0.40	0.32	0.25	0.48	−0.11	−0.28	0.53	0.63	0.15
***phi031***	10	3.40	2.27	0.82	0.61	0.50	0.74	−0.28	−0.23	0.10	0.27	0.69
***umc1075***	5	3.40	2.49	1.00	0.42	0.61	0.68	0.30	0.31	0.38	0.10	2.13
***umc1327***	5	3.20	2.17	0.82	0.20	0.47	0.69	0.39	0.57	0.70	0.31	0.56
***phi084***	6	3.40	2.34	0.85	0.51	0.50	0.52	−0.01	−0.02	0.24	0.26	0.71
***umc1786***	8	3.00	1.93	0.69	0.49	0.41	0.74	−0.23	−0.20	0.28	0.40	0.37
***p-bnlg176***	4	3.00	2.09	0.81	0.30	0.50	0.48	0.35	0.40	0.56	0.26	0.71
***umc1941***	8	3.40	2.24	0.85	0.41	0.47	0.57	0.10	0.13	0.39	0.30	0.58
**Mean**	6.70	3.18	2.11	0.78	0.40	0.46	0.63	0.10	0.12	0.41	0.32	0.70
**St. dev.**	2.263	0.38	0.30	0.16	0.15	0.09	0.11	0.31	0.33	0.22	0.14	0.54
**Entrebin**	29	2.90	2.05	0.77	0.41	0.49		0.08	0.16	0.39	0.28	0.64
**Perloz**	33	3.30	2.13	0.84	0.51	0.50		−0.02	−0.01	0.24	0.26	0.73
**Châtillon**	24	2.40	1.51	0.43	0.16	0.25		0.21	0.35	0.76	0.64	0.14
**Arnad**	35	3.50	2.39	0.91	0.44	0.53		0.13	0.17	0.35	0.22	0.87
**Arnad-Crest**	38	3.80	2.47	0.96	0.47	0.55		0.13	0.14	0.31	0.19	1.05
**Mean**	31.8	3.18	2.11	0.78	0.40	0.46		0.10	0.16	0.41	0.32	0.69
**St. dev.**	5.45	0.54	0.38	0.21	0.14	0.12		0.08	0.13	0.20	0.18	0.34

**Table 2:** List of private alleles detected in the present collection. For each allele, the corresponding landrace is reported as well as the locus, allele size and frequency.

Landrace	Locus	Allele	Frequency
Entrebin	*phi031*	196	0.475
Entrebin	*umc1786*	124	0.050
Entrebin	*umc1786*	144	0.075
Entrebin	*umc1941*	103	0.050
Perloz	*phi127*	111	0.500
Perloz	*phi031*	192	0.425
Perloz	*phi084*	149	0.050
Perloz	*umc1786*	130	0.375
Perloz	*umc1941*	96	0.125
Perloz	*umc1941*	108	0.050
Châtillon	*phi076*	142	0.125
Châtillon	*umc1401*	116	0.300
Châtillon	*phi031*	205	0.025
Châtillon	*umc1075*	142	0.300
Arnad	*umc1401*	138	0.031
Arnad	*phi031*	172	0.031
Arnad	*phi031*	209	0.031
Arnad	*phi031*	224	0.219
Arnad-Crest	*phi076*	133	0.031
Arnad-Crest	*phi076*	136	0.125
Arnad-Crest	*phi076*	144	0.094
Arnad-Crest	*phi076*	146	0.031
Arnad-Crest	*phi076*	163	0.094
Arnad-Crest	*umc1401*	143	0.125
Arnad-Crest	*phi127*	100	0.094
Arnad-Crest	*phi031*	189	0.063

**Table 3:** Nei’s genetic distance between the different maize landraces.

Entrebin	Perloz	Châtillon	Arnad	Arnad-Crest	
0.000					Entrebin
0.711	0.000				Perloz
0.855	1.063	0.000			Châtillon
0.657	0.666	0.707	0.000		Arnad
0.900	0.613	0.663	0.353	0.000	Arnad-Crest

**Table 4:** Pairwise F_ST_ between the different maize landraces.

Entrebin	Perloz	Châtillon	Arnad	Arnad-Crest	
0.000					Entrebin
0.197	0.000				Perloz
0.326	0.343	0.000			Châtillon
0.197	0.194	0.287	0.000		Arnad
0.229	0.181	0.269	0.117	0.000	Arnad-Crest

## Data Availability

Not applicable.

## Supplementary Materials

The following supporting information can be downloaded at: https://www.mdpi.com/article/10.3390/plants12142674/s1, Figure S1: PCoA of the five corn landraces based on averaged data of populations. Supplementary Material S1 PCoA 3D of the 92 individuals of the maize landraces.
